# Systemic induction of phosphatidylinositol-based signaling in leaves of arbuscular mycorrhizal rice plants

**DOI:** 10.1038/s41598-020-72985-6

**Published:** 2020-09-28

**Authors:** Sonia Campo, Blanca San Segundo

**Affiliations:** 1 Centre for Research in Agricultural Genomics (CRAG) CSIC-IRTA-UAB-UB, Campus Universitat Autònoma de Barcelona (UAB), Bellaterra (Cerdanyola del Vallés), Barcelona, Spain; 2 Consejo Superior de Investigaciones Científicas (CSIC), Barcelona, Spain

**Keywords:** Plant molecular biology, Plant symbiosis

## Abstract

Most land plants form beneficial associations with arbuscular mycorrhizal (AM) fungi which improves mineral nutrition, mainly phosphorus, in the host plant in exchange for photosynthetically fixed carbon. Most of our knowledge on the AM symbiosis derives from dicotyledonous species. We show that inoculation with the AM fungus *Funneliformis mosseae* stimulates growth and increases Pi content in leaves of rice plants (*O. sativa,* cv Loto, ssp *japonica*). Although rice is a host for AM fungi, the systemic transcriptional responses to AM inoculation, and molecular mechanisms underlying AM symbiosis in rice remain largely elusive. Transcriptomic analysis identified genes systemically regulated in leaves of mycorrhizal rice plants, including genes with functions associated with the biosynthesis of phospholipids and non-phosphorus lipids (up-regulated and down-regulated, respectively). A coordinated regulation of genes involved in the biosynthesis of phospholipids and inositol polyphosphates, and genes involved in hormone biosynthesis and signaling (jasmonic acid, ethylene) occurs in leaves of mycorrhizal rice. Members of gene families playing a role in phosphate starvation responses and remobilization of Pi were down-regulated in leaves of mycorrhizal rice. These results demonstrated that the AM symbiosis is accompanied by systemic transcriptional responses, which are potentially important to maintain a stable symbiotic relationship in rice plants.

## Introduction

Arbuscular mycorrhizal fungi (AMF or AM fungi) are soil-borne obligate biotrophs that form mutualistic associations with most terrestrial plants^1–6^. The fungus provides the host plant with nutrients, mainly phosphorus, which generally improves plant growth and fitness. In return, the host plant provides the AM fungus with sugars and lipids, both representing a major source of organic carbon in the fungus^7^. In this symbiosis, the fungus penetrates and colonizes the root cortex, forming differentiated symbiotic structures, known as arbuscules, which are the sites for nutrient exchange between the two organisms. Besides plant nutrition improvement, AM fungi often confer tolerance to biotic and abiotic stresses^8–14^. The molecular mechanisms of interactions between plants and AM fungi have mainly been investigated using the model legumes of *Medicago truncatula* and *Lotus japonicus*^15^.

Rice is one of the most important cereal crops in the world and a staple for more than half of the global population. Rice is also a host for AM fungi, and several studies have found positive effects of AM fungi on growth, pathogen resistance, and drought tolerance in rice plants^16^. Even though rice can establish symbiotic relationships with AM fungi, the molecular and physiological responses to inoculation with an AM fungus in rice plants remain less explored. The main reason for this backwardness is that rice is mostly grown under flooded conditions (paddy fields), and plants growing in aquatic environments have been traditionally considered to be non-mycorrhizal. However, natural colonization with AM fungi has been reported in rice plants under aerobic and flooded conditions^17,18^.

Root colonization by AM fungi involves drastic reprogramming of gene expression for the control AM symbiosis. Most studies focused on transcriptional responses in roots of mycorrhizal plants^19–22^, whereas transcriptional responses in leaves of mycorrhizal plants have not yet been systematically investigated. Most AM-responsive genes in roots are involved in transcriptional regulation (e.g., transcription factors) and signal transduction (e.g., components of the “common symbiosis signaling” pathway), as well as in nutrient transport, or sugar and lipid transport^15,23^. Phytohormones are also important regulators of AM symbiosis^6,24,25^.

This study aimed to investigate systemic transcriptional alterations in leaves of mycorrhizal rice plants. These studies were carried out on the rice cultivar Loto (*Oryza sativa*, ssp. *japonica*) interacting with the AM fungus *Funneliformis mosseae*. Evidence is presented that the AM symbiosis triggers coordinated regulations in the expression of genes involved in lipid signaling (phospholipids and non-phosphorus lipids), hormone biosynthesis and signaling (jasmonic acid, ethylene), and genes involved in Pi homeostasis. These results provided a global view of genes, and members of gene families, that will advance our understanding on mechanisms underlying the AM symbiosis in rice.

## Results

### Effect of *F. mosseae* inoculation on growth of rice plants

**Figure 1:**
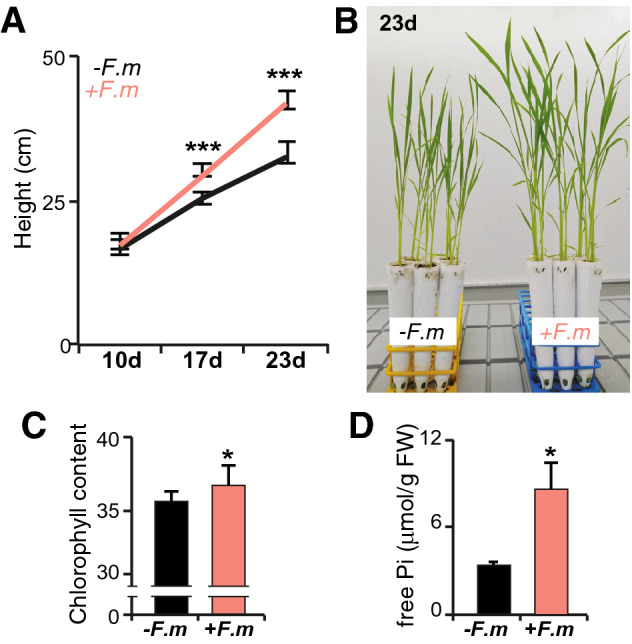
Phenotype of rice (*Oryza sativa* spp. *japonica*, cv Loto) inoculated with the AM fungus *F. mosseae*. (**A**) Rice plants were inoculated with *F. mosseae* (+ *F.m*; red line), or non-inoculated (-*F.m*; black line). Height of Loto rice plants was determined at the indicated times after inoculation with *F. mosseae*. Data are mean ± SE (n = 10). Asterisks denote statistical differences (*p < 0.05, ANOVA test; *F. mosseae*-inoculated *vs* non-inoculated). (**B**) Appearance of Loto rice plants at 23 days post-inoculation (dpi). (**C**) Chlorophyll content in leaves of mycorrhizal and non-mycorrizal plants at 23 dpi. Data are mean ± SE (n = 10). (**D**) Pi content in leaves of mycorrhizal and non-mycorrhizal Loto plants at 23 dpi. Data are mean ± SE (n = 5, each biological replicate is a pool of 2 individual leaves). *FW* fresh weight.

**Figure 2:**
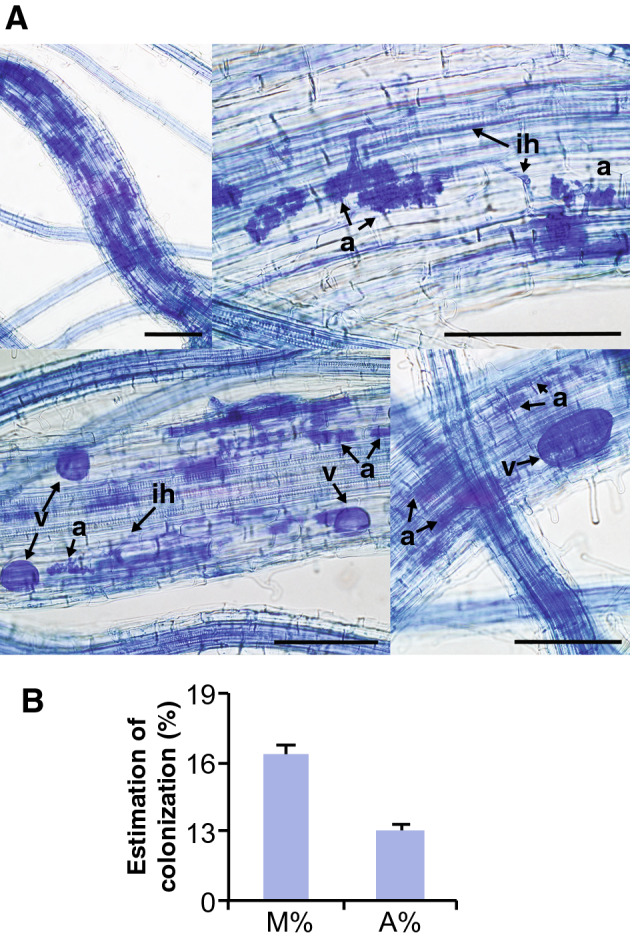
Colonization by *F. mosseae* in roots of rice (cv Loto) plants. (**A**) Representative cotton blue staining of roots colonized by *F. mosseae* at 8 wpi. Light micrographs show intracellular hyphae (ih), arbuscules (a) and vesicles (v). Scale bar = 100 μm. (**B**) Estimation of root colonization was determined by the Trouvelot method at 8 weeks post-inoculation. M%, Intensity of the mycorrhizal colonization in the root system; A%, Arbuscule abundance in the root system.

It is known that rice cultivars differ widely for colonization with the AM fungus *R. irregularis*^26^. In this work, we observed that inoculation with the AM fungus *F. mosseae* has an important effect on growth of Loto rice plants (Fig. 1A). *F. mosseae*-inoculated plants grew faster than non-inoculated plants, the stimulatory effect on plant growth being observed as early as 17 days after *F. mosseae* inoculation (Fig. 1A,B). Chlorophyll content, and indicator of the physiological status of plants, also increased in mycorrhizal plants (Fig. 1C). Inoculation with *F. mosseae* significantly increased Pi content in leaves of Loto plants (Fig. 1D), thus, supporting the formation of a functional AM symbiosis. Microscopic observations of cotton blue-stained roots confirmed root colonization by *F. mosseae*. Typical AM fungal structures, such as hyphae, vesicles and arbuscules were clearly observed in roots of *F. mosseae*-inoculated Loto plants (Fig. 2).

### Transcriptome analysis reveals the impact of AM symbiosis in rice leaves

**Figure 3:**
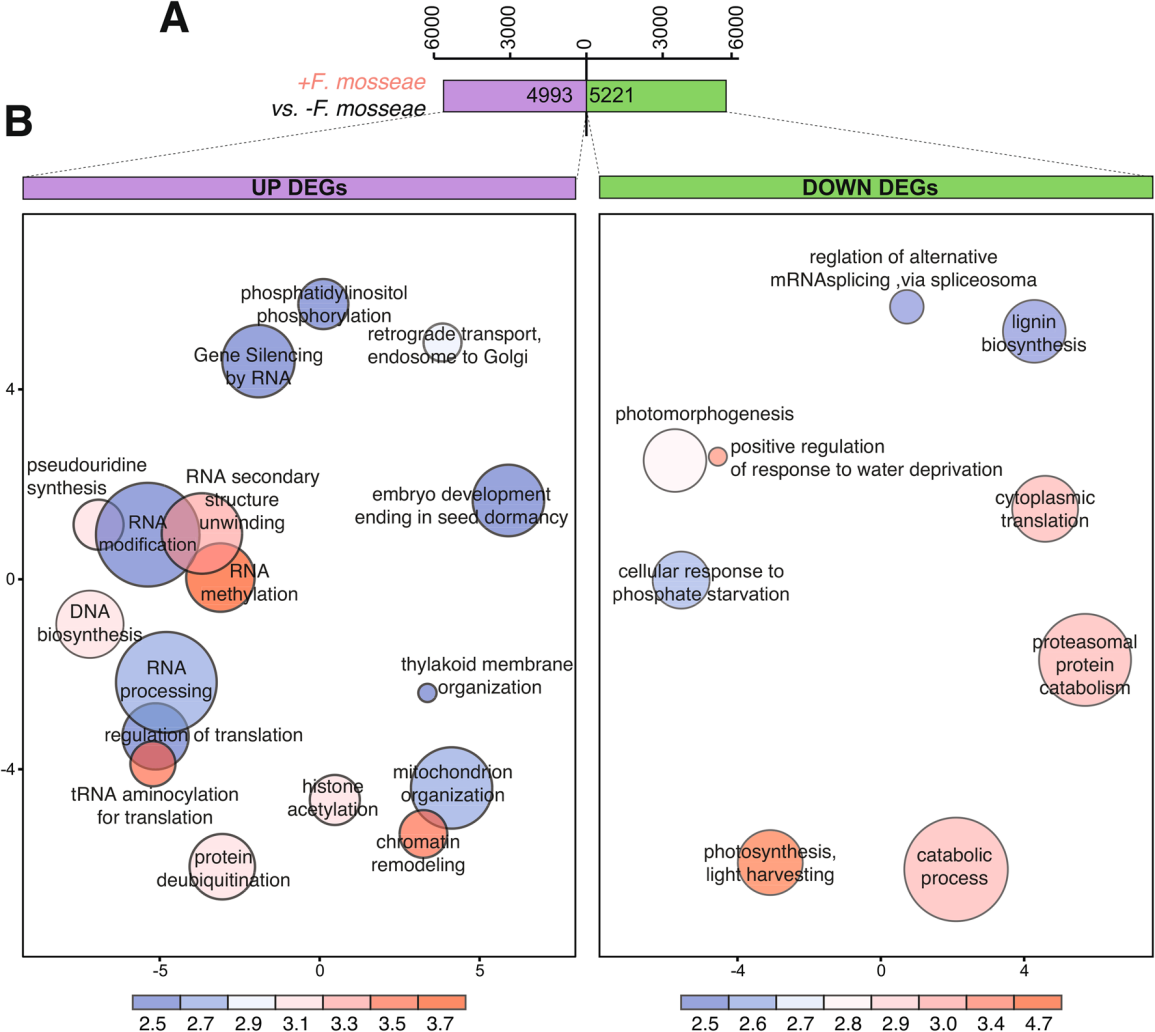
Differentially expressed genes (DEGs) in leaves of *F. mosseae*-inoculated rice plants relative to non-inoculated plants by RNA-seq analysis. Leaves of 30-day old rice plants (cv Loto) were collected after 23 dpi of root inoculation with the AM fungus *F. mosseae* (+ *F. mosseae*). No inoculum was added to non-inoculated plants (-*F. mosseae*). (**A**) Number of DEGs (+ *F. mosseae vs* -*F. mosseae*). Up-regulated (log2 fold change [FC] ≥ 0.5; purple) and down-regulated (log2FC ≤ -0.5; green) genes (p < 0.05, false discovery rate [FDR] < 0.01, n = 3). (**B**) GO terms enriched in up- (left panel) and down- (right panel) regulated DEGs. The most enriched GO terms were identified (number of DEG ≥ 10; Enrichment Score ≥ 2.5) and visualized using REVIGO after reducing redundancy and clustering of similar GO terms (https://revigo.irb.hr/). Disc color (blue to red) represent the degree of GO enrichment (*Enrichment Score*) and disc size is proportional to the frequency of the GO term in the GO *Oryza sativa* database (larger and smaller discs represent more general and more specific terms, respectively). Full list of genes in enriched GO terms is presented in Supplementary Table S2.

To investigate the molecular mechanisms underlying AM symbiosis in rice, we performed transcriptome analysis of leaves from mycorrhizal and non-mycorrhizal plants. Differentially expressed genes (DEGs) were identified based on significance level (FDR ≤ 0.01) and log2 fold change (FC) as the threshold for up-regulated (FC ≥  + 0.5) and down-regulated (FC ≤—0.5) genes. Using these criteria, 4993 and 5221 genes were found to be up-regulated and down-regulated, respectively, in leaves of mycorrhizal plants relative to non-inoculated plants (Fig. 3A; Supplementary Fig. S1A,B; Supplementary Table S1). Principal component analysis revealed a clear separation between non-inoculated and *F. mosseae*-inoculated plants (Supplementary Fig. S1C). These results support that the AM symbiosis has a strong impact on the leaf transcriptome of Loto rice plants. Similarly, massive transcriptional changes were reported in rice roots during AM symbiosis^19^.

Gene Ontology (GO) enrichment analysis of Biological Process (Supplementary Table S2) revealed that GO annotations related to “Phosphatidylinositol phosphorylation” were over-represented in the set of up-regulated genes in leaves of mycorrhizal plants (Fig. 3B, left panel). The GO categories of “Gene silencing”, “RNA modification and RNA processing”, and “Regulation of translation”, were also enriched among the up-regulated genes (Fig. 3B, left panel).

A significant enrichment in the GO term of “Cellular response to phosphate starvation” occurred in the set of down-regulated DEGs (Fig. 3B, right panel). Other GO categories enriched among the down-regulated genes were “Proteasomal protein catabolism”, or “Catabolic processes”. Results obtained by RNA-Seq analysis were validated by qRT-PCR (Supplementary Fig. S2A, B). A good correlation between qRT-PCR and RNA-seq data was observed (Supplementary Fig. S2C).

### Transcriptional regulation of phosphoinositide and inositol polyphosphate biosynthesis genes in mycorrhizal rice plants

**Figure 4:**
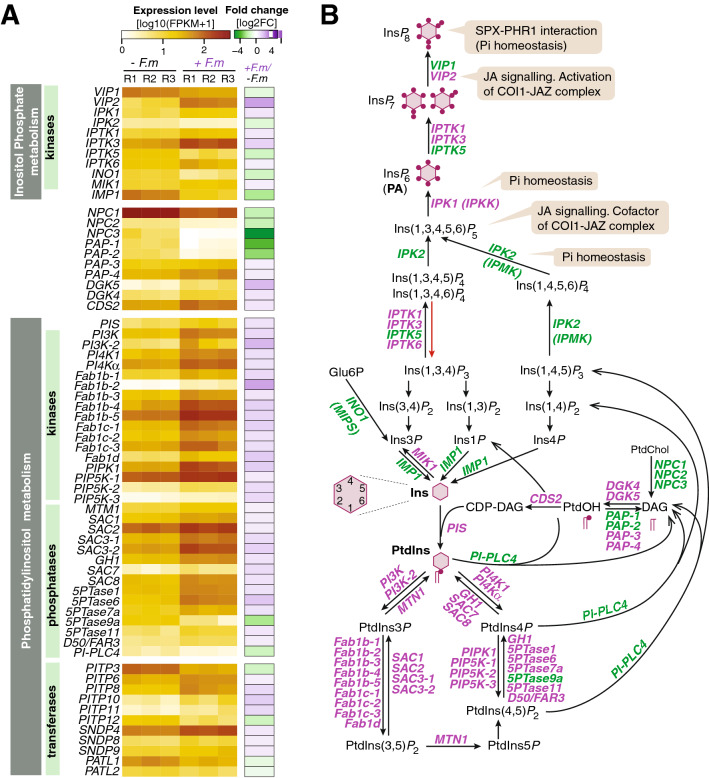
Differential expression of genes involved in inositol polyphosphates (Ins*P*) and phosphoinositides (PtdIns) metabolism in leaves of rice (cv Loto) plants. RNA-Seq was used to identify DEGs in leaves of *F. mosseae*-inoculated vs non-inoculated (− *F.m *vs − *F.m*) rice at 23 dpi. (**A**) Heatmaps showing expression level (left panel; log10 [FPKM + 1]) and Fold Change (right panel; log2FC) of DEGs. Gene expression is represented from pale yellow (less expressed) to orange (more expressed). Upregulated (log2FC ≥ 0.5; purple) and downregulated (log2FC ≤ − 0.5; green) DEGs. Data represented are individual replicates (n = 3, each biological replicate (R) consisting in a pool of 6 leaves from individual plants). The full gene names and ID list are in listed in Table 1 and Supplementary Table S3. (**B**) Biochemical pathways for the synthesis of Ins*P* and PtdIns*P*. Interactions with JA signaling and Pi homeostasis are indicated. DEGs in leaves of AM-inoculated *vs* leaves of non-inoculated rice plants are indicated in purple (up-regulated) and green (down-regulated). Phosphoinositides species identified in plants include PtdIns monophosphates (PtdIns3*P*, PtdIns4*P,* PtdIns5*P*), and PtdIns biphosphates (PtdIns(3,5)*P*_2_, PtdIns(4,5)*P*_2_). The presence of PtdIns(3,4)*P*_2_ or PtdIns(3,4,5)*P*_3_ in plants has not been reliable confirmed. Enzyme abbreviations are as follows: *5PTase* Phosphatidylinositol 5-phosphatase, *CDS* Cytidine diphosphate diacylglycerol synthase, *D50* DWARF50, *CDP-DAG* Cytidine diphosphate diacylglycerol, *DAG* diacylglycerol, *DGK* Diacylglycerol kinase, *FAB* Phosphatidylinositol 3phosphate 5-kinase, *GH1* Grain number and plant Height1, *IMP* Inositol monophosphatase, *Ins* myo-inositol (the positions on the inositol ring are designated 1–6), *IPKK* Inositol-pentakisphosphate 2-kinase, *IPMK* Inositol polyphosphate multikinase, *IPTK* Inositol 1,3,4-trisphosphate 5/6-kinase, *MIK* Myo-inositol kinase, *MTM* Phosphatidylinositol 3-phosphatase myotubularin, *NPC* Non-specific phospholipase C, *PA* phytic acid, *PAP* Phosphatidic acid phosphatase, *PATL* Patellin Sec14-domain, *PI3K* Phosphatidylinositol 3-kinase, *PI4K* Phosphatidylinositol 4-kinase, *PIP5K* Phosphatidylinositol 4-phosphate 5-kinase, *PI-PLC* Phosphatidylinositol-specific phospholipase, *PIS* Phosphatidylinositol synthase, *PITP* Phosphatidylinositol-transfer proteins, *PtdChol* Phosphatidylcholine, *PtdIns* Phosphatidylinositol, *PtdOH* Phosphatidic acid, *VIP* VIP/PPIP5K diphosphoinositol pentakisphosphate kinase, *SAC* Phosphatidylinositol 3,5-bisphosphate 5-phosphatase and Phosphatidylinositol 4-phosphate phosphatase, *SNDP* Sec14-nodulin domain-containing protein.

As previously mentioned, GO analysis revealed that genes involved in “Phosphatidylinositol phosphorylation” were significantly enriched in leaves of mycorrhizal rice. In plants, Phosphatidylinositol (PtdIns) (or phosphoinositide, PI) is an essential phospholipid that is required for the production of both membrane-bound phosphoinositides and soluble inositol polyphosphates (InsPs, or IPs). Besides their roles as membrane building blocks, phosphoinositides also exert regulatory functions through interaction with a wide range of cellular proteins^27^. As for inositol polyphosphates, they function as cofactors, regulators, and second messengers in plants. At present, however, there are major gaps in our knowledge about the possible contribution of phosphoinositides and inositol polyphosphates in interactions between plants and AM fungi. This fact prompted us to further investigate the expression of genes involved in inositol polyphosphate and phosphoinositide metabolism in mycorrhizal rice plants. To note, we found that the expression of phosphoinositide and inositol polyphosphate biosynthesis was highly regulated in leaves of mycorrhizal rice plants (Fig. 4A; Table 1; Supplementary Table S3). The mycorrhiza-regulated genes in the phosphoinositide and inositol polyphosphate biosynthesis pathway identified in this study are presented in Fig. 4B.

**Table 1:** DEGs genes involved in the biosynthesis of Phosphatidylinositol and Inositol polyphosphates in leaves of *F. mosseae*-inoculated rice plants.

Gene name	Gene ID	Gene product	EC number	FC
*VIP1*	Os01g0777700	Diphosphoinositol-pentakisphosphate kinase (PPIP5K)	2.7.4.24	− 0.96
*VIP2*	Os03g0689100			2.57
*IPK1*	Os04g0661200	Inositol-pentakisphosphate 2-kinase (IPKK)	2.7.1.158	0.86
*IPK2*	Os02g0523800	Inositol polyphosphate multikinase (IPMK)	2.7.1.151; 2.7.1.140	− 1.13
*IPTK1*	Os10g0103800	Inositol 1,3,4-trisphosphate 5/6-kinase (IPTK)	2.7.1.159	0.80
*IPTK3*	Os03g0726200			1.37
*IPTK5*	Os10g0576100			− 1.54
*IPTK6*	Os09g0518700			0.61
*INO1*	Os03g0192700	Inositol-3-phosphate synthase 1 (MIPS)	5.5.1.4	− 1.52
*MIK1*	Os07g0507300	Myo-inositol kinase (MIK)		0.55
*IMP1*	Os03g0587000	Inositol monophosphatase (IMP)	3.1.3.- ; 3.1.3.25	− 2.37
*NPC1*	Os03g0826600	Non-specific phospholipase (NPC)	3.1.4.3	− 1.84
*NPC2*	Os01g0955000			− 1.80
*NPC3*	Os03g0852800			− 5.50
*PAP-1*	Os01g0666000	Phosphatidate phosphatase (PAP)	3.1.3.4	− 3.73
*PAP-2*	Os05g0549900			− 2.91
*PAP-3*	Os08g0359200			0.55
*PAP-3*	Os09g0308900			0.66
*DGK5*	Os03g0425300	Diacylglycerol kinase (DGK)	2.7.1.107	2.04
*DKK4*	Os12g0576900			0.88
*CDS2*	Os01g0758400	Cytidine diphosphate diacylglycerol synthase (CDS)	2.7.7.41	0.83
*PIS*	Os06g0492000	Phosphatidylinositol synthase (PIS)	2.7.8.11	0.93
*PI3K*	Os05g0180600	Phosphatidylinositol 3-kinase (PI3K)	2.7.1.137	1.24
*PI3K-2*	Os08g0307400			2.07
*PI4K1*	Os11g0209700	Phosphatidylinositol 4-kinase (PI4K)	2.7.1.67	1.04
*PI4Kα*	Os03g0711200			1.00
*Fab1b-1*	Os03g0399532	Phosphatidylinositol 3phosphate 5-kinase (FAB/PIKFYVE)	2.7.1.150	1.21
*Fab1b-2*	Os04g0691900			2.48
*Fab1b-3*	Os08g0104700			0.88
*Fab1b-4*	Os08g0450700			1.40
*Fab1b-5*	Os08g0450800			1.32
*Fab1c-1*	Os06g0259000			1.39
*Fab1c-2*	Os08g0428900			0.96
*Fab1c-3*	Os09g0402300			1.06
*Fab1d*	Os12g0236700			1.58
*PIPK1*	Os03g0701800	Phosphatidylinositol 4-phosphate 5-kinase (PIP5K)	2.7.1.68	1.53
*PIP5K-1*	Os02g0822500			0.70
*PIP5K-2*	Os03g0356582			1.14
*PIP5K-3*	Os07g0658700			1.21
*MTM1*	Os08g0556700	Phosphatidylinositol 3-phosphatase myotubularin-1 (MTM)	3.1.3.64; 3.1.3.95	0.60
*SAC1*	Os08g0109100	Phosphatidylinositol 3,5-bisphosphate 5-phosphatase (SAC)	3.1.3.	0.68
*SAC2*	Os03g0182400			0.68
*SAC3-1*	Os02g0782600			1.12
*SAC3-2*	Os06g0195600			1.36
*GH1*	Os02g0554300	Phosphatidylinositol 4-phosphate phosphatase (SAC)		0.94
*SAC7*	Os11g0309000			1.15
*SAC8*	Os03g0290500			0.96
*5PTase1*	Os01g0716800	Phosphatidylinositol 5-phosphatase	3.1.3.36	1.03
*5PTase6*	Os03g0663700			1.65
*5PTase7a*	Os05g0489000			1.01
*5PTase9a*	Os03g0238300			− 2.42
*5PTase11*	Os03g0626500			0.83
*D50*	Os02g0477700			0.68
*PI-PLC4*	Os05g0127200	Phosphatidylinositol‐specific phospholipase C (PI-PLC)	3.1.4.11	− 1.62
*PITP3*	Os01g0926800	Sec14-like phosphatidylinositol transfer proteins		− 1.48
*PITP6*	Os02g0321500			0.55
*PITP8*	Os02g0752000			1.19
*PITP10*	Os05g0267800			1.54
*PITP11*	Os05g0267100			1.27
*PITP12*	Os03g0850700			− 1.62
*SNDP4*	Os02g0133100			0.80
*SNDP8*	Os02g0721800			0.66
*SNDP9*	Os08g0497300			0.69
*PATL1*	Os05g0429400			− 0.90
*PATL2*	Os01g0874700			− 0.62

The EC (Enzyme Commission) number for each enzyme is indicated.

*FC* log2 Fold Change.

Among the genes up-regulated in leaves of mycorrhizal plants there were genes involved in: (i) the synthesis of PtdIns, namely *cytidine diphosphate-diacylglycerol *(*CDP-DAG*) *synthase* (*OsCDS2*), and *PtdIns synthase* (*OsPIS*); (ii) kinase genes acting in the production of phosphorylated derivatives of PtdIns, either PtdIns monophosphates [PtdIns3*P*, PtdIns4*P*] (PI3K and PI4K family proteins), or PtdIns bisphosphates [PtdIns(3,5)*P*_2_, PtdIns(4,5)*P*_2_] (FAB1/PIKFYVE and PIP5K family proteins) (Fig. 4A,B; Table 1). Genes encoding PtdIns3*P* and PtdIns4*P* phosphatases, and PtdIns(3,5)*P*_2_ (Myotubularin, SAC1, and 5´phosphatases family proteins) were also up-regulated. The production of Ins3*P* from Glucose-6P appears to be repressed in leaves of mycorrhizal plants, as revealed by the down-regulation of *myo-inositol-1-phosphate synthase* (*INO1 *or* MIPS*) (Fig. 4B). Altogether these findings point to fine-tuning of phosphoinositide levels in mycorrhizal rice.

In plants, InsPs are synthesized via two pathways: the lipid-independent (or soluble) and lipid–dependent pathways^28^. In the lipid-independent pathway, soluble inositol is phosphorylated through the action of kinases, whereas in the lipid-dependent pathway, different InsP intermediates originate through degradation of PtdIns by the action of phosphoinositide-specific phospholipases^28^. Here, the unique pathway that appears to be activated in leaves of mycorrhizal plants is the soluble pathway, as revealed by the up-regulation the *myo-inositol kinase* (*MIK1*) and down-regulation of the phosphoinositide-specific phospholipase C (*PI-PLC4*) (Fig. 4A,B; Table 1). Also, the expression of genes encoding kinases responsible for the production of the different inositol polyphosphates was found to be regulated in leaves of mycorrhizal plants (Fig. 4A,B; Table 1). They were: *OsIPK1* (*Inositol-pentakisphosphate 2-kinase*, *IPPK*), involved in the phosphorylation of Ins(1,3,4,5,6)*P*_5_ to produce Ins(1,2,3,4,5,6)*P*_6_ (phytic acid, or InsP_6_), *OsIPK2* (*Inositolpolyphosphate multikinase*, *IPMK),* involved in sequential phosphorylations from inositol triphosphates Ins(1,4,5)*P*_3_] to inositol-pentakisphosphate Ins(1,3,4,5,6)*P*_5_] and *OsIPTK1, OsIPTK3, OsIPTK5, OsIPTK6* (Inositol 1,3,4-trisphosphate 5/6-kinase, IPTK), involved in phosphorylation of the 5/6-position of inositol triphosphates Ins(1,3,4)*P*_3_] to produce inositol-tetrakisphosphate Ins(1,3,4,5)*P*_4_ and Ins(1,3,4,6)*P*_4_ and Ins(1,3,4)*P*_3_. *OsIPTK1, OsIPTK3, OsIPTK5* have InsP_6_ kinase activity in vitro, and produce Ins*P*_7_ and Ins*P*_8_ from phytic acid^29^*.* Also, *OsVIP1* and *OsVIP2*, encoding inositol hexakisphosphate and diphosphoinositol-pentakisphosphate kinase Vip1/PPIP5K-like proteins, produce Ins*P*_7_ and Ins*P*_8_. VIP2, the rice homolog for the Arabidopsis VHI2 protein, critical for Ins*P*_8_ production^30^, was upregulated in leaves of mycorrhizal rice plants.

Collectively, results here presented support that the inoculation of rice roots with the AM fungus *F. mosseae* results in systemic alterations in the expression of genes involved in the biosynthesis of inositol polyphosphates and phosphoinositides. It is worth mentioning that although several studies described a correlation between inositol phosphate metabolites and Pi homeostasis in the model dicotyledonous Arabidopsis^30–34^, at present, no systematic study of phosphoinositide or inositolpolyphosphate signaling has been performed in AM symbiosis in cereal species, as rice is. Similarly, cross-talk between inositol polyphosphates and JA signaling has been described in Arabidopsis^35–37^. Taking into account that inositol phosphate metabolites act as important signaling molecules, the observed mycorrhiza-induced alterations in the expression of genes involved in inositol phosphate metabolic pathways is expected to have an impact on multiple signaling processes in mycorrhizal rice plants, including Pi homeostasis and hormonal regulation. Along with this, genes involved in hormone signaling were also found to be regulated in leaves of mycorrhizal rice plants, as described below.

### Expression of genes related to hormone signaling in leaves of mycorrhizal rice plants

In addition to developmental regulation, plant hormones also play important roles in symbiotic interactions between plants and AM fungi^38^. Each of the plant hormones possesses specific functions and they also interact with each other either in an antagonistic or cooperative way. However, contradictory results have been reported in studies related to hormone signaling in AM symbiosis, such as Jasmonic acid (JA), ethylene (ET), Salicylic acid (SA) and auxins, which led to the notion that these regulatory mechanisms are dependent on the plant species and growth conditions (e.g., Pi supply conditions)^38^. Contrary to what was previously described in *Medicago truncatula* and tomato, the establishment of AM symbiosis appears to occur independently of Jasmonic Acid in rice^39^.

**Figure 5:**
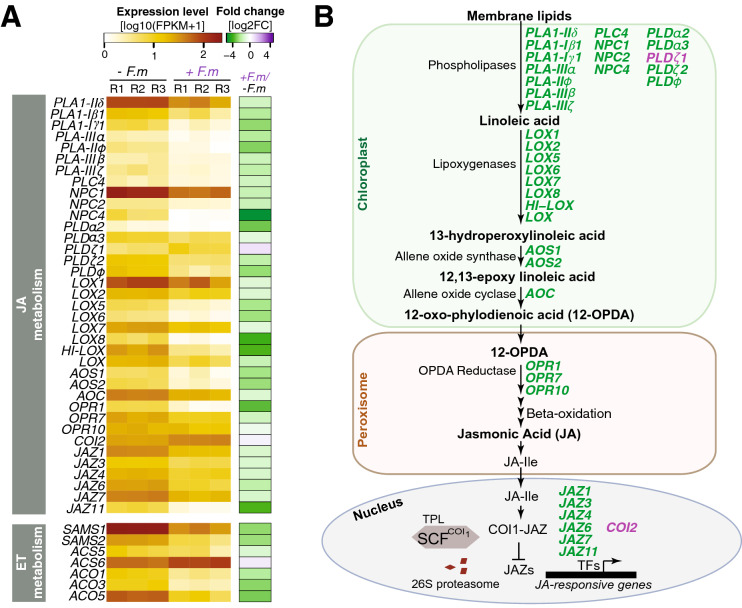
Differential expression of genes involved in biosynthesis of Jasmonic Acid and Ethylene in leaves of rice (cv Loto) plants. RNA-Seq was used to identify DEGs in leaves of *F. mosseae*-inoculated *vs* non-inoculated (+ *F.m vs* − *F.m*) plants at 23 dpi. (**A**) Heatmaps showing RNA-seq expression level and fold change as indicated in Fig. 4A legend. The full gene names and ID list are in listed in Supplementary Table S4. (**B**) Biochemical pathway for the synthesis of Jasmonic acid (JA). DEGs in leaves of AM-inoculated rice plants are indicated in purple (up-regulated) and green (down-regulated). Enzyme abbreviations are as follows: *AOC* Allene oxide cyclase, *AOS* Allene oxide synthase, *COI* Coronatine insensitive 1, *HPL3* Hydroperoxide lyase 3, *JAZ* jasmonate ZIM-domain protein, *LOX* Lipoxygenase, *NPC* Non-specific phospholipase C, *OPR* 12-oxo-phylodienoic acid, *PLA* Phospholipase A, *PLC* Phospholipase C, *PLD* Phospholipase D, *SCF* SKp1, Cullin and F-box proteins, *TPL* TOPLESS protein.

Transcriptome analysis revealed a down-regulation of many genes involved in JA biosynthesis and signaling in leaves of mycorrhizal plants (Fig. 5A; Supplementary Table S4). The mycorrhiza-regulated genes in the JA biosynthesis pathway identified in this study are presented in Fig. 5B. Although most of our knowledge in JA biosynthesis and signaling comes from studies in Arabidopsis, all homologs of Arabidopsis genes have been identified in rice^40^. JA (and JA-derivatives) originates from lipids, preferentially α–linolenic acid, which is released from galactolipids of chloroplast membranes. Linoleic acid is then converted to OPDA through the enzymes Lipoxygenase (LOX), allene oxidase synthase (AOS), and allene oxide cyclase (AOC) in the chloroplast. OPDA is then transported to peroxisomes where it is reduced by OPDA reductase (OPR), and after three rounds of β-oxidation JA is produced. To note, among the mycorrhiza down-regulated genes in rice leaves, we identified genes involved in the different steps of JA biosynthesis, namely *Phospholipases A* (*PLA1-IIδ, PLA1-Iβ1, PLA1-Iγ1, pPLA-IIIα, pPLA-IIϕ, pPLA-IIIβ, pPLA-IIIζ), Phospholipases C (PLC4, NPC1, NPC2, NPC3), Phospholipases D* (*PLDα2, PLDα3, PLDζ2, PLDφ*), *Lipoxygenases* (*OsLOX1, OsLOX2, OsLOX5, OsLOX6, OsLOX7, OsLOX8, OsHI-LOX, OsLOX*), *AOS* (*OsAOS1, OsAOS2*), *AOC* (represented by a unique member in the rice genome), and *OPR* (*OsOPR1, OsOPR7*, *OsOPR10*) genes (Fig. 5A,B; Supplementary Table S4). Although the functional implication of this negative regulation of JA biosynthesis genes remains to be investigated, these findings suggest that JA biosynthesis is repressed in leaves of mycorrhizal rice plants which is consistent with previous observations indicating that JA biosynthesis is dispensable for AM symbiosis in rice^39^.

Regarding JA signaling, it is known that perception of JA-Ile (the bioactive JA derivative) by CORONATINE INSENSITIVE 1 (COI1) and JASMONATE ZIM DOMAIN (JAZ) proteins triggers the degradation of JAZ proteins by the SCF^COI1^ complex-mediated 26 proteasome for activation of downstream JA-responsive genes (Fig. 5). Whereas the Arabidopsis genome has a single *COI1* gene (*COI1*), the rice genome contains three closely related *COI* genes (*OsCOI1*, *OsCOI2*, *OsCOI3*). The three *COI* genes play a role in JA signaling in rice by interacting with JAZ proteins in rice^41^. Our transcriptome analysis revealed up-regulation of *OsCOI2*, whereas 6 *OsJAZ* genes (*OsJAZ1, OsJAZ3, OsJAZ4, OsJAZ6, OsJAZ7, OsJAZ11*) were repressed in leaves of mycorrhizal plants (Fig. 5; Supplementary Table S4).

Among the set of down-regulated genes in leaves of mycorrhizal rice, we also identified the three key genes involved in ethylene biosynthesis, namely the *S-adenosyl-L-methionine synthetase* (*OsSAMS1, OsSAMS2*), which catalyzes the conversion of S-adenosylmethionine (AdoMet) from methionine, the *1-aminocyclopropane-1-carboxylic acid* (ACC) synthases (*OsACS5*) which catalyzes the conversion of AdoMet to ACC, the rate-limiting step in ET biosynthesis, and ACC oxidases (*OsACO1, OsACO3, OsACO5*) which catalyzes oxidation of ACC to form ethylene (Fig. 5A; Supplementary Table S4). Although not proven, this observation points to the repression of ethylene biosynthesis in rice leaves during AM symbiosis.

### Expression pattern of genes involved in Pi signaling and homeostasis

**Figure 6:**
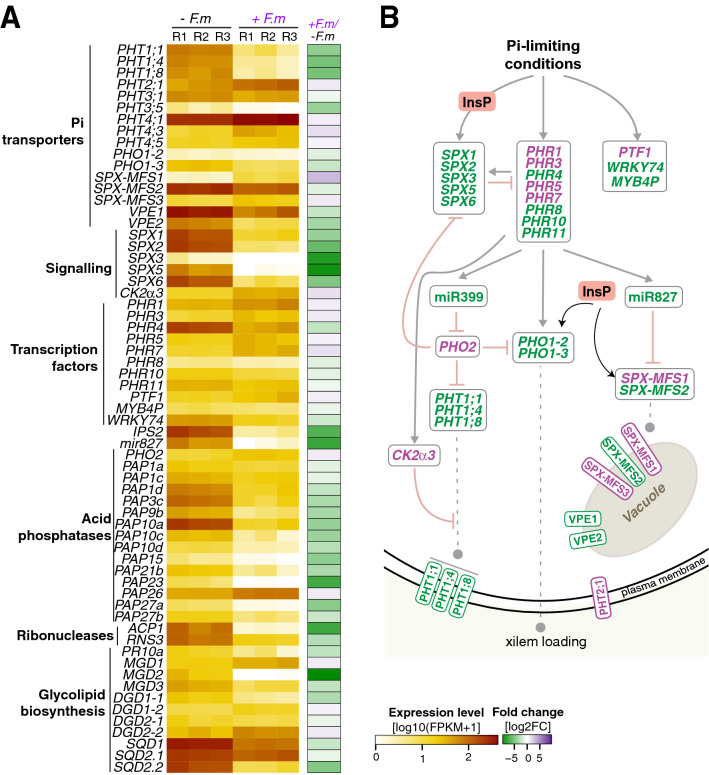
Differential expression of genes involved in Pi signaling and homeostasis in leaves of rice (cv Loto) plants. RNA-Seq was used to identify DEGs in leaves of *F. mosseae*-inoculated vs non-inoculated (+ *F.m* vs − *F.m*) plants at 23 dpi. (**A**) Heatmap showing RNA-seq expression level and fold change as indicated in Fig. 4A legend. The full gene names and ID list are in listed in Supplementary Table S5. (**B**) Regulatory network of Pi starvation responses**.** The PHR and related transcription factors are negatively regulated by the SPX proteins, which can sense Inositol polyphosphates (InsPs). Under P limitation, PHR relieves negative post-transcriptional control of Pi transporters responsible of Pi uptake through the induction of *MIR399* (targeting *PHO2*). In rice, miR827 targets *SPX-MFS1* and *SPX-MSF2* transcripts. The SPX-MFS1 and SPX-MSF2 proteins, as well as the Vacuolar Efflux Proteins (VPEs) modulate Pi homeostasis by regulating Pi influx and efflux from vacuoles. Under sufficient Pi-conditions, the CK2α3/β3 kinase phosphorylates PHT1 resulting in retention of PHT1 transporters in the endoplasmic reticulum. InsPs are sensed by the SPX domain of SPX-EXS proteins (e.g., PHO1-2, PHO1-3) and SPX-MFS proteins. Most of these studies have been done in Arabidopsis^43^.

As previously mentioned, genes in the “Cellular Response to Phosphate starvation” GO term were highly represented among down-regulated DEGs in leaves of mycorrhizal rice plants (Fig. 3B, right panel). This category included genes that mediate Pi signaling, transport, and remobilization. In particular, the expression of an important number of phosphate transporters was found to be down-regulated. They included: members of the *PHT* phosphate transporters belonging to the *PHT1* (*OsPHT1;1, PHT1;4, OsPHT1;8*), *PHT2* (*OsPHT2;1*), *PHT3* (*OsPHT3;1, OsPHT3;5*) and *PHT4* (*OsPHT4;1, OsPHT4;3, OsPHT4;5*) families, and *PHOSPHATE1* (*PHO1*) transporters (*OsPHO1-2, OsPHO1-3*) (Fig. 6A; Supplementary Table S5). In Arabidopsis, phosphorylation of PHT1 phosphate transporters by CK2α3/β3 kinase negatively affects trafficking to the plasma membrane from the endoplasmic reticulum^42^. We noticed that the CK2α3 subunit gene was up-regulated in leaves of mycorrhizal rice pointing to a possible retention of PHT1 transporters in the ER (Fig. 6A; Supplementary Table S5). Figure 6B represents the signaling events involved in Pi homeostasis altered in mycorrhizal rice plants.

PHR1 (PHOSPHATE STARVATION RESPONSE 1), a member of the MYB superfamily of transcription factors, has been shown to control the transcriptional activation and repression responses to phosphate starvation in plants^43–46^ In particular, PHR1 and related transcription factors are known to be key players in Pi signaling in Arabidopsis and rice^43,45,46^. To note, the expression of several PHR transcription factors were regulated in leaves of mycorrhizal plants (Fig. 6; Supplementary Table S5). Of them, *OsPHR4* was strongly down-regulated, indicating that this particular *PHR* family member might have a specific function in regulating Pi homeostasis in leaves of mycorrhizal rice. Other transcription factors that are involved in the phosphate starvation responses were also regulated in leaves of mycorrhizal rice, such as *OsMYB4P* and *OsWRKY74* (down-regulated), or PTF1 (up-regulated) (Fig. 6; Supplementary Table S5)^47–49^. Furthermore, the expression of several SPX domain-containing protein genes was repressed in leaves of mycorrhizal plants (*OsSPX1, OsSPX2, OsSPX3, OsSPX5, OsSPX6)*. SPX domain-containing proteins are known to function as a sensor for Pi level in controlling Pi homeostasis^50,51^.

Pi import and export from the vacuole, the primary intracellular compartment for Pi storage in plant cells, serve as a critical mechanism for the maintenance of appropriate cytosolic Pi levels. In rice, three members of SPX proteins possess an MFS (Major Facilitator Superfamily) domain, and are involved in Pi transport across the tonoplast, SPX-MSF1, SPX-MSF2, and SPX-MSF3. An opposite behavior in the expression of these genes could be observed in leaves of mycorrhizal rice plants (down-regulation of *OsSPX-MSF2*; up-regulation of *OsSPX-MSF1* and *OsSPX-MSF3*). In this respect, Pi deficiency was reported to induce (*OsSPX-MSF2*) or repress (*OsSPX-MSF1*, *OsSPX-MSF3*) the expression of these genes in rice^51^. Expression of the vacuolar Pi efflux transporters *OsVPE1* and *OsVPE2* was also down-regulated in leaves of mycorrhizal rice (Fig. 6A,B; Supplementary Table S5).

Genes that have proven to be implicated in the remobilization of Pi from organic P resources, such as purple acid phosphatases (*PAPs*), acid phosphatase (*ACP1*), and ribonucleases (*RNS3, PR10a*) were repressed in leaves of mycorrhizal rice plants, indicating that these plants have an adequate P status (Fig. 6A; Supplementary Table S5). In this respect, it is well known that membrane phospholipids (a major source for internal Pi supply) are degraded under Pi limiting conditions, which are then replaced by non-phosphorus lipids (galactolipids and sulfolipids). Galactolipids are essential for a functional photosynthetic apparatus in the chloroplast, and include monogalactosyldiacylglycerol (MGDG) and digalactosyldiacylglycerol (DGDG) lipids, which are synthesized by MGDG synthase (or MGD) and DGDG synthase (or DGD). Plants have evolved various acclimation responses to cope with phosphate depletion, including Pi remobilization from endogenous phosphorus-containing resources like phospholipids^52^. Thereby membrane phospholipids are dephosphorylated and can be used as an internal phosphate source, while galactolipids are incorporated into the membrane to maintain membrane functionality. Remarkably, the expression of genes involved in the biosynthesis of these major galactolipids, MGDG (*OsMGD1*, *OsMGD2*, *OsMGD3*) and DGDG (*OsDGD1-1*, *OsDGD1-2*, *OsDGD2-1*, *OsDGD2-2*) was regulated in leaves of mycorrhizal rice, their expression being mainly down-regulated in leaves of mycorrhizal rice (Fig. 6; Supplementary Table S5). These observations suggest that membrane lipid remodeling toward galactolipids, most probably, does not occur in leaves of mycorrhizal plants because of the greater capacity of mycorrhizal roots to take up Pi. On the other hand, the synthesis of sulfolipids is catalyzed by uridine diphosphate-sulfoquinovose synthase (encoded by *OsSQD1*) and sulfoquinovosyldiacylglycerol synthase (*OsSQD2*). The expression of *OsSQD1*, *OsSQD2.1* and *OsSQD2.2* was repressed in leaves of mycorrhizal rice.

**Figure 7:**
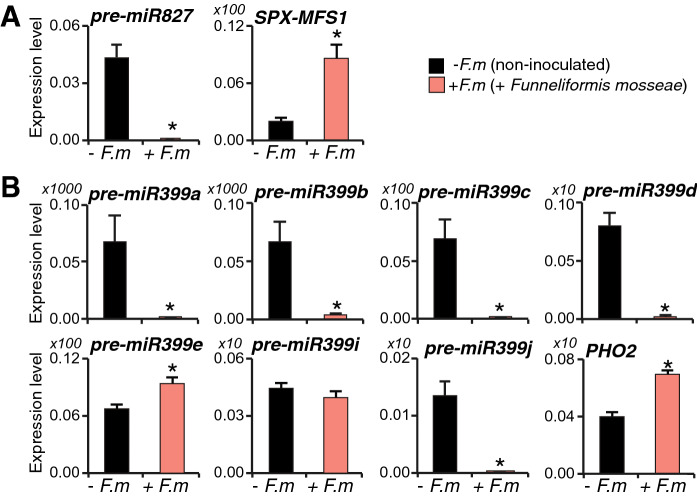
Accumulation of miR399 and miR827 precursor transcripts in leaves of mycorrhizal plants. RNA was obtained from leaves of non-inoculated (− *F.m*) and AM inoculated (+ *F.m*) rice (cv. Loto) plants at 23 dpi. The expression level was determined by qRT-PCR. Primers used sequences are listed in Supplementary Table S6. Data are mean ± SE (n = 3; each sample consisted of a pool of 3 individual leaves; *p < 0.05, ANOVA test). (**A**) Accumulation of precursor transcripts for *miR827* and its target genes *OsSPX-MFS1.* (**B**) Accumulation of precursor transcripts for *miR399* family members and its target genes *OsPHO2.*

On the other hand, the implication of the microRNAs miR399 and miR827 in regulating Pi starvation responses in plants is well documented^50,53^. Our transcriptome analysis revealed down-regulation of miR827, and up-regulation of its target gene *SPX-MSF1* encoding a vacuolar Pi transporter (Fig. 6; Supplementary Table S5), which was further confirmed by qRT-PCR and statistical analysis of miR827 precursor transcripts and *SPX-MSF1* (Fig. 7A). miR827 transcripts were barely detectable in mycorrhizal rice. Thus, a miR827-mediated cleavage of *SPX-MSF1* transcripts would regulate the intracellular distribution of Pi between the cytoplasm and vacuoles^50,51^.

On the other hand, the rice miRNA399 family consists of 11 members (miRNA399a to miR399k; miRBASE v22.1) (Supplementary Fig. S3), all of then targeting the *PHOSPHATE2* (*OsPHO2,* also named *LTN*, *Leaf Tip Necrosis*) gene, which encodes a ubiquitin-conjugating E2 enzyme that mediates the degradation of Pi transporters^54,55^. As for most rice miRNAs, no RAP-DB gene ID is currently assigned to any of the miR399 family members, and, accordingly, this miRNA was not identified as DEG in our RNA-seq analysis. Nevertheless, the miR399 target *OsPHO2* was significantly up-regulated in leaves of mycorrhizal rice (Fig. 6; Supplementary Table S5). Accordingly, the expression of each miR399 member was analyzed by qRT-PCR. Precursor transcripts for seven out of the eleven miR399 family members (*MIR399a, MIR399b, MIR399c, MIR399d, MIR399e, MIR399i, MIR399j*) accumulated in leaves of non-mycorrhizal control plants, these plants being grown under low Pi supply (Fig. 7A). Of them, *MIR399a, MIR399b, MIR399c, MIR399d, MIR399j* were barely detectable in leaves of mycorrhizal plants. Conversely, *MIR399e* and *MIR399i* precursor transcripts accumulated at higher or similar level, respectively, in mycorrhizal plants compared with non-mycorrhizal plants. These results demonstrated a statistically significant differential regulation (from repression to activation) of miR399 family members in leaves of mycorrhizal rice plants. In this study, precursor transcripts for *MIR399f, MIR399g*, and *MIR399h* were undetectable. As expected and in accordance with RNA-seq data, anticorrelation between *MIR399* and *OsPHO2* occurred in leaves of mycorrhizal rice (Fig. 7B).

Overall, comparative analysis of leaf transcriptomes of mycorrhizal and non-mycorrhizal rice plants revealed systemic regulation of distinct members of genes involved in Pi signaling and homeostasis. Repression of phosphate starvation-responsive genes and those involved in the biosynthesis of non-phosphorus membrane lipids is consistent with an improved Pi nutrition, as revealed by the observation that mycorrhizal Loto plants have a higher Pi content in leaves than non-mycorrhizal plants (Fig. 1D).

## Discussion

Although rice is the staple food of more than half of the world's population, very few studies focused on the impact of root colonization by AM fungi on rice. Here, we show that inoculation with *F. mosseae* stimulates growth and increases Pi content in leaves of rice plants (cv Loto), supporting that Loto plants are effectively colonized by this AM fungus. We also show that root colonization by *F. mosseae* is associated to massive systemic transcriptional alterations in the rice leaf transcriptome. The transcriptional responses in leaves of mycorrhizal rice plants appear to differ substantially from those previously reported in roots of rice plants colonized by *R. irregularis*^19,56^. Here, alterations in the expression of genes related to mineral transport were described in lateral roots, whereas secondary cell wall and phytohormone metabolism genes are regulated in adult crown roots^19^. As AM symbiosis improves Pi nutrition with the potential to optimize rice production, a deeper understanding of transcriptional regulatory mechanisms underlying AM symbiosis in rice is a requisite to ultimately use this symbiotic association in rice cultivation while avoiding the adverse impact of chemical fertilizers.

**Figure 8:**
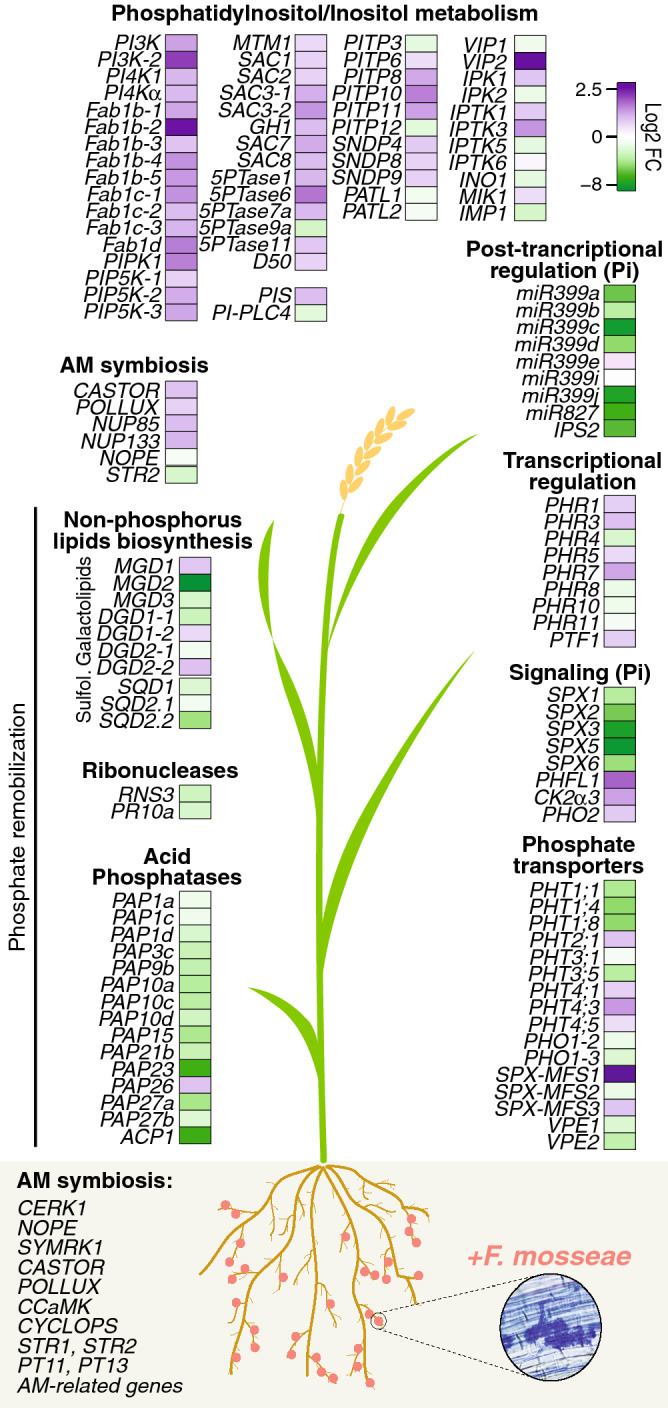
Summary of systemically regulated genes in leaves of mycorrhizal rice plants. Genes involved in phosphatidylinositol biosynthesis and phospholipid-based signaling events are indicated. Heatmaps showing log2 fold change (AMF-inoculated vs non inoculated) rice (cv. Loto) plants are included. Up-regulated (purple) and down-regulated (green) DEGs. The expression of genes involved in AM symbiosis has been mostly studied in roots of mycorrhizal plants^57^. In this study, several AM symbiosis-related genes were also found to be up-regulated in leaves of mycorrhizal rice plants, such as two cation channels *OsCASTOR (Os03g0843600) and OsPOLLUX (Os01g0870100),* and two nuclear porins *OsNUP85 (Os01g074620) and OsNUP133 (Os03g022550)*.

Diverse processes were regulated in leaves of mycorrhizal rice plants, such as those related to phospholipid and hormone signaling, as well as regulatory mechanisms controlling Pi homeostasis (summarized in Fig. 8). Reprogramming of host gene expression in leaves is potentially important to maintain an appropriate Pi status in the plant and a stable symbiotic relationship.

Results here presented revealed systemic alterations in the expression of genes involved in phosphatidylinositol metabolism and signaling, including major phospholipids (phosphoinositides), inositol polyphosphates, and non-phosphorus lipids (galactolipids and sulfolipids). Whereas most genes involved in phosphoinositide and inositol polyphosphate biosynthesis were up-regulated in leaves of mycorrhizal rice, genes involved in the biosynthesis of non-phosphorus lipids (galactolipids and sulfolipids) were essentially down-regulated. Studies in Arabidopsis have shown that plants adapt to low P status by replacing phospholipids by galactolipids and sulfolipids. Accordingly, non-phospholipid biosynthesis is induced while phospholipid biosynthesis is repressed in Pi-starved plants^58,59^. Our transcriptome analysis showed activation of phospholipid biosynthesis genes (Fig. 4) and repression of non-phosphorus lipid biosynthesis genes (Fig. 6) in leaves of mycorrhizal rice plants (summarized in Fig. 8). These findings suggest that inoculation with *F. mosseae* provides an adequate Pi level to the rice plant and that Pi acquired in AM colonized roots is used efficiently. Regulation of phospholipid biosynthesis genes reinforces the notion that phospholipids might play a central role in the AM symbiosis.

Transcript profiling also revealed up-regulation of kinase genes involved in phosphatidylinositol biosynthetic processes. Plant phosphoinositides and inositol polyphosphates are formed via phosphorylation of the phospholipid PtdIns by the activity of specific kinases. Dynamic changes in phosphoinositide levels occur in plant tissues in which enzymes mediating biosynthesis and degradation of phosphoinositides are continuously synthesized and degraded^27^. With this in mind, it is not surprising to find that kinase and phosphatase genes regulating a particular reaction opposite one another are both up-regulated. In comparison to enzymes of phosphatidylinositol biosynthesis, however, there is only limited information about phosphatases and/or phospholipases involved in phosphatidylinositol degradation. Whereas biochemical assays can only reveal global kinase activity, transcript profiling allowed us to discriminate among members of the same family that are specifically regulated in leaves of mycorrhizal rice (e.g., IPTKs or inositol kinases).

As phospholipids are one of the main structural components of membranes, alterations in the expression of phospholipid biosynthesis genes might influence membrane structure and/or function. But phospholipids and inositol polyphosphates are also important signaling molecules that function as cofactors of partner proteins. It is tempting to hypothesize that alterations in phospholipid biosynthesis genes are accompanied by changes in the level of these metabolites, which would then have an impact on diverse cellular processes. However, the functional implications of alterations in phospholipid biosynthesis genes in AM symbiosis in rice have yet to be clarified. It will now be of interest to investigate whether mycorrhiza-induced alterations in phospholipids have a consequence in long-distance signaling. In this respect, the presence of phospholipids and phospholipid-binding proteins, in the phloem of several plant species has long been reported^60–62^. A phospholipid-based signaling mechanism might then operate during AM symbiosis.

Moreover, alterations in inositol polyphosphate biosynthesis genes might have an effect on hormonal signaling processes, particularly with regard to JA signaling. In favor of this hypothesis, Ins*P*_5_ and Ins*P*_4_ were found to interact with the COI/JAZ complex involved in JA perception in Arabidopsis^36^. In other studies, Ins*P*_7_ and Ins*P*_8_ were reported to promote the binding between COI1 and JAZ1 for the regulation of JA-dependent responses^30^. The *VIP* gene functioning in phosphorylation of PP-IP_5_ (named as *VIH2* in Arabidopsis) regulates the synthesis of Ins*P*_8_, the cofactor of the COI-JAZ receptor complex^30^. Results here presented, together with those obtained by other authors, broadens our view on the existence of cross-talk between phospholipid metabolism and JA signaling processes during AM symbiosis. Clearly, many efforts are still needed to understand the connections between these signaling pathways in AM symbiosis.

Plants associate with AM fungi when grown under low Pi soil conditions and, accordingly, AM symbiosis is generally studied in plants grown in low Pi. In line with this, down-regulation of Pi starvation-responsive genes occurs in leaves of mycorrhizal rice compared with non-mycorrhizal plants (the later ones responding to the situation of low Pi supply). Among the down-regulated genes in leaves of mycorrhizal rice, we identified genes belonging to different families of Pi-responsive genes. They included genes involved in: (i) Pi transport (e.g. several *PHT,* vacuolar transporters; *PHO1*); (ii) signaling processes (*SPX domain*, *CK2α3*, *PHO2*); (iii) transcriptional regulation (*PHR*, *PTF1* transcription factors); (iv) post-transcriptional regulation (miR399, miR827); (v) Pi recycling (phosphatases, RNases); and (vi) non-phosphorus membrane lipid biosynthesis (galactolipids, sulpholipids). Pi starvation-responsive genes whose expression is regulated in leaves of mycorrhizal rice are summarized in Fig, 8. A differential regulation of genes belonging to each one of these families points to a specialization of these mycorrhiza-regulated family members in controlling Pi homeostasis in rice leaves. As an example, a functional specialization of miR399 species in regulating Pi homeostasis in leaves of mycorrhizal plants can be anticipated. Also, down-regulation of genes involved in Pi starvation responses or Pi remobilization from macromolecules (PAPs, RNases, galactolipid and sulfolipid biosynthesis genes) further supports that rice plants have a normal level of Pi.

As an additional level of complexity in the regulation of Pi homeostasis in plants, there is growing evidence on a regulatory role of inositol polyphosphates in Pi signaling^33^. Indeed, the SPX domain was recently defined as a Pi sensor in Arabidopsis plants that regulates Pi transport activity of SPX domain-containing proteins through binding inositol polyphosphate signaling molecules^32,33^. Additionally, the Arabidopsis PHR1 and rice PHR2 transcription factors, which are recognized as central components of the phosphate starvation response, have been shown to interact with SPX proteins^63,64^. The interaction between SPX proteins and the AtPHR1 transcription factor results in PHR1 inactivation, being this interaction promoted by inositol polyphosphates^34^. Results here presented demonstrated connections between inositol polyphosphate metabolism and Pi homeostasis in AM symbiosis. Furthermore, mechanisms involved in Pi homeostasis and hormonal signaling must be tightly regulated in leaves of mycorrhizal rice, which would then allow the host plant to maximize the benefits of Pi acquisition during association with the AM fungus. Additional studies are, however, needed to determine the exact mechanisms by which phospholipids might exert a regulatory function in maintaining Pi homeostasis and how this would affect hormonal processes in mycorrhizal rice plants.

In conclusion, our work provided new insights on the systemic alterations in gene expression during the symbiotic association of rice plants with an AM fungus. Phosphorus deficiency is widespread in all major rice ecosystems and is the major growth-limiting factor in acid upland soils where soil P-fixation capacity is often high (https://www.knowledgebank.irri.org). To circumvent this problem, P fertilizers are applied extensively in rice farming to improve plant performance, but their use has negative effects on the environment. Although there are still numerous issues that still need to be addressed, pieces of evidence here presented might accelerate research on the AM symbiosis in rice, which will open new avenues for improvement of sustainability in rice production. A more sustainable way to improve mineral nutrition in rice would be through the use of AM fungi, which would also avoid the overuse of chemical fertilizers.

## Materials and methods

### Plant and fungal material

The rice cultivar Loto (*Oryza sativa* ssp *japonica*) was used in this study. Loto rice is a heritage variety of short-grain rice widely cultivated in northern Italy (the first rice producer in Europe), also grown in southern Switzerland (the northern-most rice-growing region in Europe) and northern United States (Vermont State), making Loto a suitable rice variety for rice farming in northern latitudes.

The AMF fungus *Funneliformis mosseae* (formerly *Glomus mosseae*; FR140) was purchased from MycAgro (Bretenière, France; https://www.mycagrolab.com/) in a granular form composed of mineral inert solid particles (clay, zeolite) and *F. mosseae* propagules at a concentration of minimum 10 propagules/gram.

### Inoculation with *F. mosseae* and growth of AMF-inoculated plants

Rice Loto seeds were dehusked, surface-sterilized twice with 5% sodium hypochlorite for 15 min, and washed extensively with sterile water. Seeds were germinated on Petri dishes with sterile water for 7 days and then transplanted to 150 ml-cones (20.5 cm; 2 plants/cone) containing a mix of 63.3% quartz sand (0.3–0.8 mm), 31.6% soil (turface and vermiculite 2:1), and 5% of granular inoculum of *Funneliformis mosseae*. No inoculum was added to the substrate for the non-inoculated, control plants. Transplanted seedlings were then grown under aerobic conditions at the greenhouse under a 14 h/10 h day/night cycle, 28 ºC/25 ºC, and 60% humidity for the required time. Rice seedlings were bottom-watered during the whole experiment. Fertirrigation was supplied at nine days after *F. mosseae* inoculation, using a modified Hoagland half-strength solution (15 ml, every two days) containing 2.5 mM KNO_3_, 2.5 mM Ca(NO_3_)_2_·4H_2_O, 1 mM MgSO_4_·7H_2_O, 0.5 mM NH_4_NO_3_, 25 μM KH_2_PO_4_, 23.15 μM H_3_BO_3_, 4.55 μM MnCl_2_·4H_2_O, 0.38 μM ZnSO_4_·7H_2_O, 0.1 μM CuSO_4_·5H_2_O, 0.14 μM Na_2_MoO_4_·2H_2_O, 26 μM Fe-EDDHA, pH 5.5)^65^.

### Analysis of root colonization

Root fragments were collected at eight weeks post-inoculation with *F. mosseae* and stained with 0.1% cotton blue in acid lactic as previously described^66^. Fifty root fragments (1 cm) per biological replicate (n = 3) were examined with a light microscope, and the intensity of root cortex colonization was determined^67^. AMF structures were examined using an Axiophot Zeiss microscope equipped with a Digital color camera (DP70 Olympus) and 40 × magnification.

### Transcriptome analysis by RNA-Seq

Total RNA was extracted from rice leaves of 30-day old plants that had been root-inoculated with *F. mosseae* (23 days post-inoculation), or not, using Maxwell(R) RSC Plant RNA Kit (Promega). Three biological replicates for each condition were examined, each biological replicate consisting of leaves from five individual plants (the youngest totally expanded leaf was selected from each individual plant). RNA concentration and purity were checked using a spectrophotometer (NanoDrop, ND-1000). RNA quality and integrity were evaluated using an Agilent 2100 Bioanalyzer (Agilent Technologies, Inc.), and only samples with an. RNA integrity number (RIN) ≥ 8 were used. RNA was analyzed by sequencing six libraries. An average of 32 736 498 clean reads/library were reported (Supplementary Table S6). Raw 125 bp paired-end reads processing and analysis were carried out as previously described^65^. RNA**‐**Seq raw data are available at the NCBI GEO database (https://www.ncbi.nlm.nih.gov/geo) under the accession number GSE148574.

Reads were mapped against the reference genome, *Oryza sativa* Japonica (IRGSP-1.0). To identify genes with significant differences in expression, an FDR cutoff < 0.01 and log2FC -0.5 ≤ or ≥ 0.5 was applied. Gene Ontology (GO) enrichment analysis (GOEA) was performed with AgriGO based on the hypergeometric test, and a minimum FDR of 0.05 was considered. Multiple testing corrections controlling false positives were also performed with the Benjamini–Hochberg method both for differential expression analysis and GOEA. (https://bioinfo.cau.edu.cn/agriGOv2/)^68^. Enriched GO terms were clustered and plotted with the online analysis tool ReviGO (https://revigo.irb.hr/)^69^.

### Expression analysis by qRT-PCR

Total RNA was extracted as described above. Quantitative RT-PCR (qRT-PCR) analyses were performed using 1 μg of total RNA and poly-dT using the High Capacity cDNA Reverse Transcription kit (Life technology, Applied Biosystems). PCR amplification was carried out using LightCycler 480 thermocycler (Roche Diagnostics, Mannheim, Germany) from 2 μl cDNA (5 ng/μl) with SYBR Green I dye and gene-specific primers (Supplementary Table S7). The rice *Ubiquitin1* (*Ubi1*) or *Ubiquitin-conjugating Enzyme 1* (*UBC1*) were used as housekeeping gene for normalization with the same results. For statistical analysis, the means and standard errors were calculated using Microsoft Excel. Significant differences were assessed using ANOVA test (*p-*value ≤ 0·05).

### Chlorophyll and Pi content

Rice plants were *F. mosseae*-inoculated or not, and grown under greenhouse conditions as described above. At 23 days after AMF inoculation, the youngest totally expanded leaves were harvested. Chlorophyll content of rice leaves was determined using a chlorophyll meter (SPAD 502 Plus Chlorophyll Meter, Spectrum Technologies) as previously described^65^. Pi content of rice leaves was determined using a colorimetric as previously described^70^.

## Supplementary information

Supplementary Figures.

Supplementary Tables.

## Data Availability

The RNA‐Seq data that support the findings of this study are available at the NCBI GEO repository (https://www.ncbi.nlm.nih.gov/geo) under the Accession Number GSE148574.

## References

[1] Parniske M (2008). Arbuscular mycorrhiza: the mother of plant root endosymbioses. Nat. Rev. Microbiol..

[2] Bonfante P, Genre A (2010). Mechanisms underlying beneficial plant–fungus interactions in mycorrhizal symbiosis. Nat. Commun..

[3] Smith SE, Read DJ (2008). Mycorrhizal Symbiosis.

[4] Choi J, Summers W, Paszkowski U (2018). Mechanisms underlying establishment of arbuscular mycorrhizal symbioses. Annu. Rev. Phytopathol..

[5] MacLean AM, Bravo A, Harrison MJ (2017). Plant signaling and metabolic pathways enabling arbuscular mycorrhizal symbiosis. Plant Cell.

[6] Müller LM, Harrison MJ (2019). Phytohormones, miRNAs, and peptide signals integrate plant phosphorus status with arbuscular mycorrhizal symbiosis. Curr. Opin. Plant Biol..

[7] Luginbuehl LH (2017). Fatty acids in arbuscular mycorrhizal fungi are synthesized by the host plant. Science.

[8] Begum N (2019). Role of arbuscular mycorrhizal fungi in plant growth regulation: implications in abiotic stress tolerance. Front. Plant Sci..

[9] Campos-Soriano L, García-Martínez J, Segundo BS (2012). The arbuscular mycorrhizal symbiosis promotes the systemic induction of regulatory defence**-**related genes in rice leaves and confers resistance to pathogen infection. Mol. Plant Pathol..

[10] Wang Y-Y, Yin Q-S, Qu Y, Li G-Z, Hao L (2018). Arbuscular mycorrhiza-mediated resistance in tomato against *Cladosporium fulvum* -induced mould disease. J. Phytopathol..

[11] Pozo MJ, Azcón-Aguilar C (2007). Unraveling mycorrhiza-induced resistance. Curr. Opin. Plant Biol..

[12] Gallou A, Lucero Mosquera HP, Cranenbrouck S, Suárez JP, Declerck S (2011). Mycorrhiza induced resistance in potato plantlets challenged by *Phytophthora infestans*. Physiol. Mol. Plant Pathol..

[13] Rivero J, Álvarez D, Flors V, Azcón-Aguilar C, Pozo MJ (2018). Root metabolic plasticity underlies functional diversity in mycorrhiza-enhanced stress tolerance in tomato. New Phytol..

[14] Fiorilli V (2018). Omics approaches revealed how arbuscular mycorrhizal symbiosis enhances yield and resistance to leaf pathogen in wheat. Sci. Rep..

[15] Wipf D, Krajinski F, Tuinen D, Recorbet G, Courty P (2019). Trading on the arbuscular mycorrhiza market: from arbuscules to common mycorrhizal networks. New Phytol..

[16] Ruiz-Sánchez M, Aroca R, Muñoz Y, Polón R, Ruiz-Lozano JM (2010). The arbuscular mycorrhizal symbiosis enhances the photosynthetic efficiency and the antioxidative response of rice plants subjected to drought stress. J. Plant Physiol..

[17] Vallino M, Greppi D, Novero M, Bonfante P, Lupotto E (2009). Rice root colonisation by mycorrhizal and endophytic fungi in aerobic soil. Ann. Appl. Biol..

[18] Bernaola L (2018). Natural colonization of rice by arbuscular mycorrhizal fungi in different production areas. Rice Sci..

[19] Gutjahr C (2015). Transcriptome diversity among rice root types during asymbiosis and interaction with arbuscular mycorrhizal fungi. Proc. Natl. Acad. Sci. USA.

[20] Tian L (2019). Comparative study of the mycorrhizal root transcriptomes of wild and cultivated rice in response to the pathogen *Magnaporthe oryzae*. Rice.

[21] Siciliano V (2007). Transcriptome analysis of arbuscular mycorrhizal roots during development of the prepenetration apparatus. Plant Physiol..

[22] Watts-Williams SJ (2019). Diverse *Sorghum bicolor* accessions show marked variation in growth and transcriptional responses to arbuscular mycorrhizal fungi. Plant. Cell Environ..

[23] Pimprikar P, Gutjahr C (2018). Transcriptional regulation of arbuscular mycorrhiza development. Plant Cell Physiol..

[24] Das, D. & Gutjahr, C. Role of phytohormones in arbuscular mycorrhiza development. in *The Model Legume Medicago truncatula* 485–500 (Wiley, Hoboken, 2020). 10.1002/9781119409144.ch61

[25] Pozo MJ, López-Ráez JA, Azcón-Aguilar C, García-Garrido JM (2015). Phytohormones as integrators of environmental signals in the regulation of mycorrhizal symbioses. New Phytol..

[26] Davidson H (2019). Spatial effects and GWA mapping of root colonization assessed in the interaction between the rice diversity panel 1 and an arbuscular mycorrhizal fungus. Front. Plant Sci..

[27] Heilmann I (2016). Phosphoinositide signaling in plant development. Development.

[28] Raboy V (2009). Approaches and challenges to engineering seed phytate and total phosphorus. Plant Sci..

[29] Laha D (2019). Arabidopsis ITPK1 and ITPK2 have an evolutionarily conserved phytic acid kinase activity. ACS Chem. Biol..

[30] Laha D (2015). VIH2 regulates the synthesis of inositol pyrophosphate InsP8 and jasmonate-dependent defenses in arabidopsis. Plant Cell.

[31] Zhu J (2019). Two bifunctional inositol pyrophosphate kinases/phosphatases control plant phosphate homeostasis. Elife.

[32] Kuo HF (2018). Arabidopsis inositol phosphate kinases IPK1 and ITPK1 constitute a metabolic pathway in maintaining phosphate homeostasis. Plant J..

[33] Dong J (2019). Inositol pyrophosphate InsP8 acts as an intracellular phosphate signal in *Arabidopsis*. Mol. Plant.

[34] Jung JY, Ried MK, Hothorn M, Poirier Y (2018). Control of plant phosphate homeostasis by inositol pyrophosphates and the SPX domain. Curr. Opin. Biotechnol..

[35] Mosblech A (2008). Phosphoinositide and inositolpolyphosphate signalling in defense responses of *Arabidopsis thaliana* challenged by mechanical wounding. Mol. Plant.

[36] Sheard LB (2010). Jasmonate perception by inositol-phosphate-potentiated COI1-JAZ co-receptor. Nature.

[37] Mosblech A, Thurow C, Gatz C, Feussner I, Heilmann I (2011). Jasmonic acid perception by COI1 involves inositol polyphosphates in *Arabidopsis thaliana*. Plant J..

[38] Bedini A, Mercy L, Schneider C, Franken P, Lucic-Mercy E (2018). Unraveling the initial plant hormone signaling, metabolic mechanisms and plant defense triggering the endomycorrhizal symbiosis behavior. Front. Plant Sci..

[39] Gutjahr C, Siegler H, Haga K, Iino M, Paszkowski U (2015). Full establishment of arbuscular mycorrhizal symbiosis in rice occurs independently of enzymatic jasmonate biosynthesis. PLoS ONE.

[40] Nguyen HT, To HTM, Lebrun M, Bellafiore S, Champion A (2019). Jasmonates—the master regulator of rice development, adaptation and defense. Plants.

[41] Lee HY (2013). *Oryza sativa COI* homologues restore jasmonate signal transduction in *Arabidopsis coi1-1* mutants. PLoS ONE.

[42] Chen J (2015). The rice CK2 kinase regulates trafficking of phosphate transporters in response to phosphate levels. Plant Cell.

[43] Puga MI (2017). Novel signals in the regulation of Pi starvation responses in plants: facts and promises. Curr. Opin. Plant Biol..

[44] Zhou J (2008). OsPHR2 is involved in phosphate-starvation signaling and excessive phosphate accumulation in shoots of plants. Plant Physiol..

[45] Rubio V (2001). A conserved MYB transcription factor involved in phosphate starvation signaling both in vascular plants and in unicellular algae. Genes Dev..

[46] Guo M (2015). Integrative comparison of the role of the PHOSPHATE RESPONSE1 subfamily in phosphate signaling and homeostasis in rice. Plant Physiol..

[47] Dai X, Wang Y, Zhang W-H (2016). OsWRKY74, a WRKY transcription factor, modulates tolerance to phosphate starvation in rice. J. Exp. Bot..

[48] Yang WT (2014). Overexpression of OsMYB4P, an R2R3-type MYB transcriptional activator, increases phosphate acquisition in rice. Plant Physiol. Biochem..

[49] Yi K (2005). OsPTF1, a novel transcription factor involved in tolerance to phosphate starvation in rice. Plant Physiol..

[50] Lin S-I (2010). Complex regulation of two target genes encoding SPX-MFS proteins by rice miR827 in response to phosphate starvation. Plant Cell Physiol..

[51] Wang C (2012). Functional characterization of the rice SPX-MFS family reveals a key role of OsSPX-MFS1 in controlling phosphate homeostasis in leaves. New Phytol..

[52] Pfaff J, Denton AK, Usadel B, Pfaff C (2020). Phosphate starvation causes different stress responses in the lipid metabolism of tomato leaves and roots. Biochim. Biophys. Acta.

[53] Chiou T-J (2006). Regulation of phosphate homeostasis by microRNA in Arabidopsis. Plant Cell.

[54] Fujii H, Chiou T-J, Lin S-I, Aung K, Zhu J-K (2005). A miRNA involved in phosphate-starvation response in Arabidopsis. Curr. Biol..

[55] Hu B (2011). LEAF TIP NECROSIS1 plays a pivotal role in the regulation of multiple phosphate starvation responses in rice. Plant Physiol..

[56] Güimil S (2005). Comparative transcriptomics of rice reveals an ancient pattern of response to microbial colonization. Proc. Natl. Acad. Sci. U. S. A..

[57] Gutjahr C (2008). Arbuscular mycorrhiza-specific signaling in rice transcends the common symbiosis signaling pathway. Plant Cell.

[58] Lan P, Li W, Schmidt W (2012). Complementary proteome and transcriptome profiling in phosphate-deficient arabidopsis roots reveals multiple levels of gene regulation. Mol. Cell. Proteomics.

[59] Woo J (2012). The response and recovery of the *Arabidopsis thaliana* transcriptome to phosphate starvation. BMC Plant Biol..

[60] Hoffmann-Benning, S. Transport and function of lipids in the plant phloem. *AOCS Lipid Library, Plant Biochemistry.* (2015). https://lipidlibrary.aocs.org/chemistry/physics/plant-lipid/transport-and-function-of-lipids-in-the-plant-phloem.

[61] Guelette B, Benning U, Hoffmann-Benning S (2012). Identification of lipids and lipid-binding proteins in phloem exudates from *Arabidopsis thaliana*. J. Exp. Bot..

[62] Barbaglia AM, Tamot B, Greve V, Hoffmann-Benning S (2016). Phloem proteomics reveals new lipid-binding proteins with a putative role in lipid-mediated signaling. Front. Plant Sci..

[63] Puga MI (2014). SPX1 is a phosphate-dependent inhibitor of phosphate starvation response 1 in Arabidopsis. Proc. Natl. Acad. Sci. USA.

[64] Wang Z (2014). Rice SPX1 and SPX2 inhibit phosphate starvation responses through interacting with PHR2 in a phosphate-dependent manner. Proc. Natl. Acad. Sci. USA.

[65] Sánchez-Sanuy F (2019). Osa-miR7695 enhances transcriptional priming in defense responses against the rice blast fungus. BMC Plant Biol..

[66] Berruti A (2013). Application of laser microdissection to identify the mycorrhizal fungi that establish arbuscules inside root cells. Front. Plant Sci..

[67] Trouvelot, A. Mesure du taux de mycorhization VA d’un systeme radiculaire. Recherche de methodes d’estimation ayant une significantion fonctionnelle (1986).

[68] Du Z, Zhou X, Ling Y, Zhang ZH, Su Z (2010). AgriGO: a GO analysis toolkit for the agricultural community. Nucleic Acids Res..

[69] Supek F, Bosnjak M, Skunca N, Smuc T (2011). REVIGO Summarizes and visualizes long lists of gene ontology terms. PLoS ONE.

[70] Ames BN (1966). Assay of inorganic phosphate, total phosphate and phosphatases. Methods Enzymol..

