# Supplementary material for: Systemic induction of phosphatidylinositol-based signaling in leaves of arbuscular mycorrhizal rice plants
Source: Sci Rep. 2020 Sep 28;10:15896. doi: 10.1038/s41598-020-72985-6 (PMC7522983; doi:10.1038/s41598-020-72985-6)
Supplement: Supplementary file 1 — Supplementary Figures. [file 41598_2020_72985_MOESM1_ESM.pdf]

Supplementary Figures for:

Systemic induction of phosphatidylinositol-based signaling in leaves of arbuscular mycorrhizal rice plants

Sonia Campo and Blanca San Segundo

Supplementary Figures

Supplementary Fig. S1. Differentially expressed genes (DEGs) in leaves of *F. mosseae* –inoculated rice plants relative to non-inoculated plants (+*F. mosseae vs –F. mosseae*)
Supplementary Fig. S2. Validation of RNA-seq data by qRT-PCR.
Supplementary Fig. S3. Alignment of rice mature miR399 sequences.

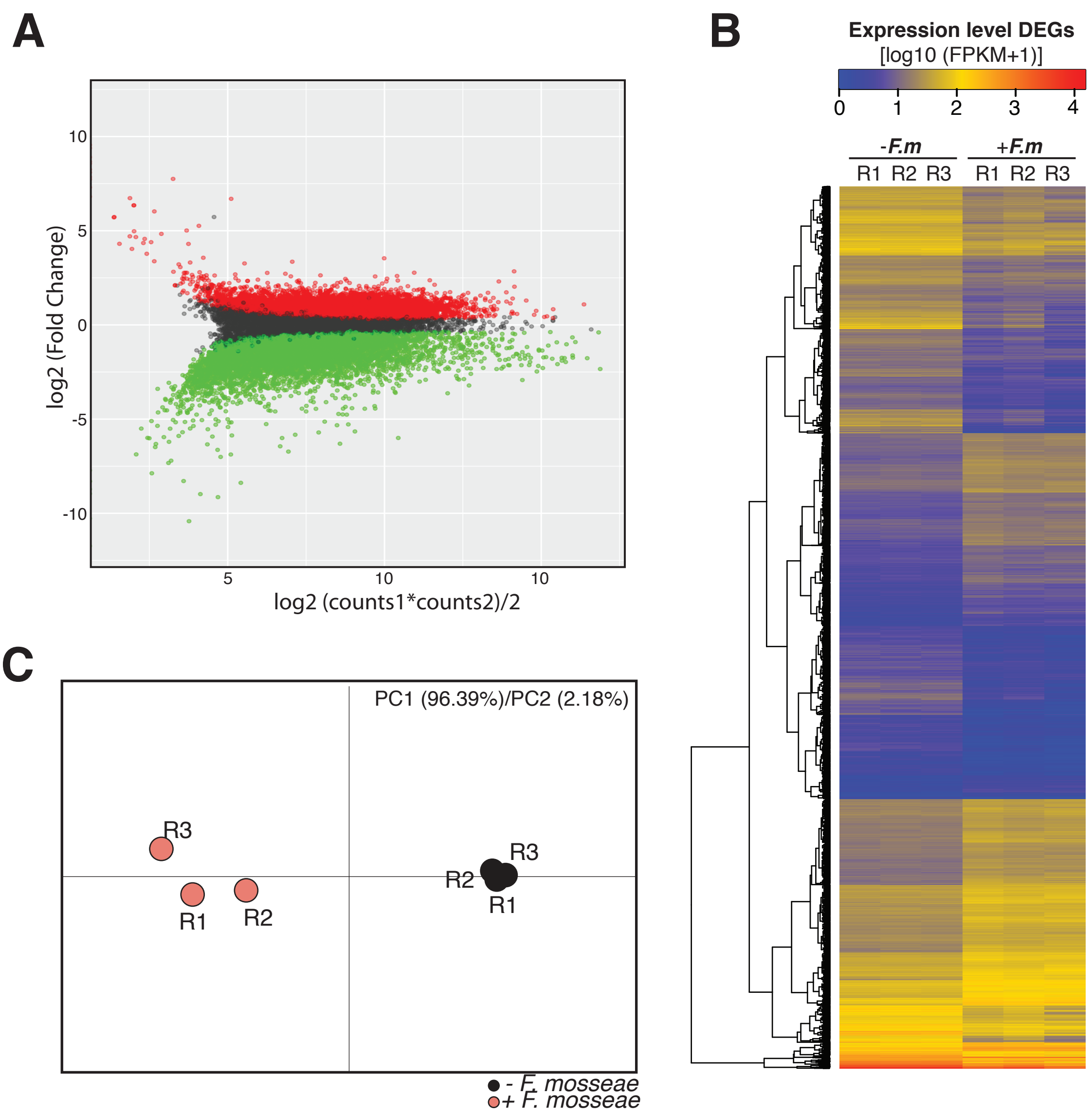

Supplementary Figure S1. Differentially expressed genes (DEGs) in leaves of *F. mosseae*-inoculated rice plants relative to non-inoculated plants (*+F. mosseae vs –F. mosseae*). Leaves of 30-day old rice plants (cultivar Loto) were collected after 23 days of root inoculation with the AM fungus *F. mosseae* (+*F.m*). No inoculum was added to the substrate for the non-inoculated, control plants (-*F.m*). **(A)** MA Plots of logarithmic fold changes (LogFCs) to average count size in RNA-seq analysis of *F. mosseae*-inoculated (+*F.m*) plants *vs* non-inoculated (-*F.m*) plants. (p <0.05, FDR <0.01, n=3). **(B)** Heatmaps showing expression level (log10 [FPKM+1]) of the DEGs in leaves of mycorrhizal plants. Gene expression is represented from blue (less expressed) to red (more expressed). Data represented are individual replicates (n=3, each biological replicate (R) consisting in a pool of 6 leaves from individual plants). The full gene names and ID list are in listed in Supplementary Table S1. Results in gene expression show homogenicity between sample replicates, that was further comfirmed in (C). **(C)** Principal component analysis (PCA) showing separation between non-inoculated (-*F.m*) and *F. mosseae*-inoculated (+*F.m*). R, biological replicate

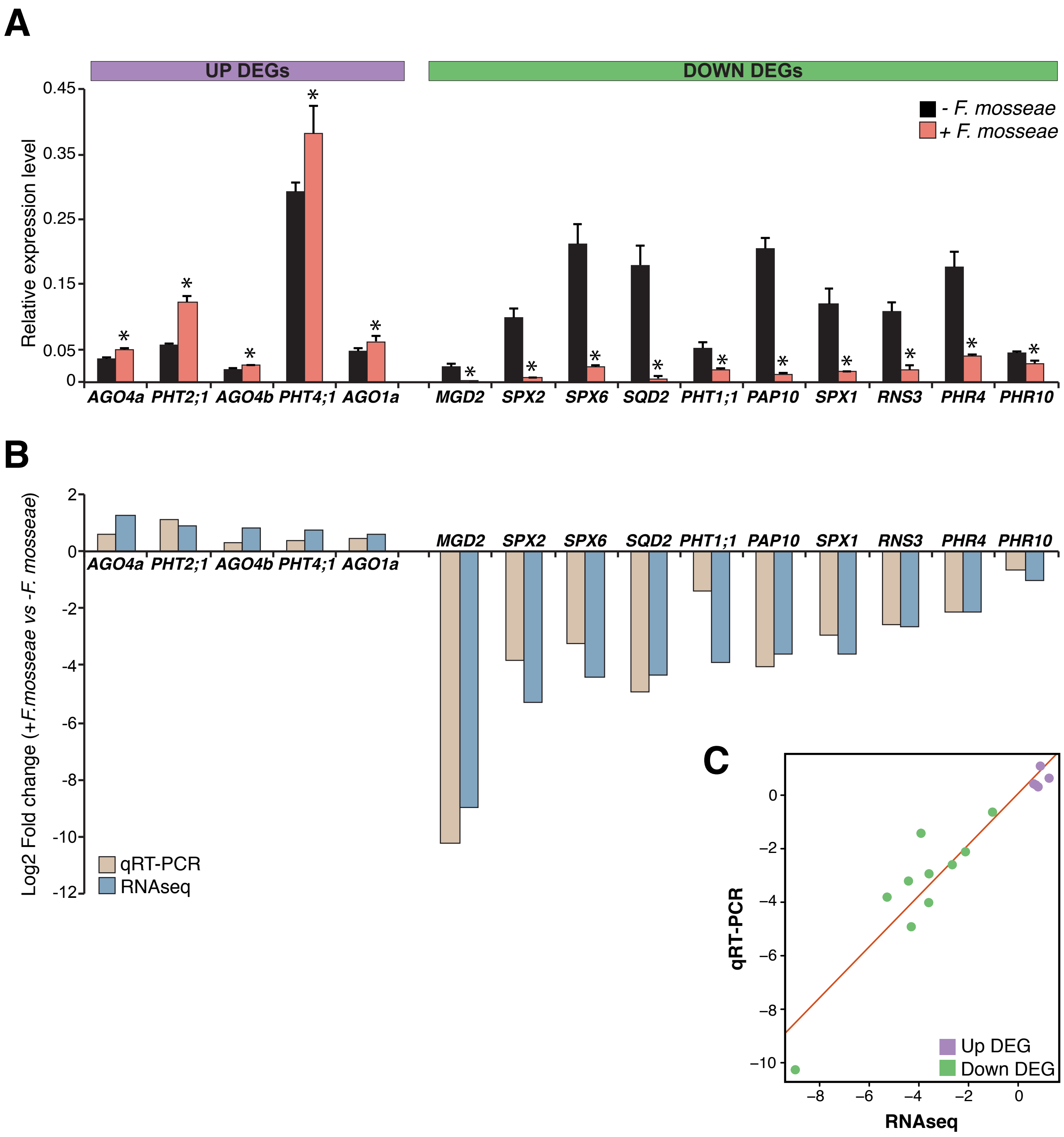

Supplementaty Figure S2. Validation of RNAseq data by qRT-PCR.
Leaves of non-inoculated (-*F.m*) and AM inoculated (+*F.m*) rice (cv Loto) plants were used (23 days after root inoculation). **(A)** Validation of RNAseq expression data as determined by qRT-PCR for the selected upregulated (purple, left panel) or downregulated (green, right panel) genes q. Data are mean ± SE (n=3) and were normalized to the rice *Ubiquitin*1. Gene-specific primers are in indicated in Supplementary Table S6 (*p < 0.05, ANOVA test, n=3). **(B)** Comparison of RNA-seq and RT-qPCR fold change values obtained for DEGs in (A). **(C)** A correlation graph of log2 fold change values from both RNAseq and RT-qPCR methods indicate that qRT-PCR and RNAseq are significantly correlated with a Pearson correlation coefficient of 0.95 and a p-value of 9.668e-08 of 15 genes.

```
osa-miR399a  UGCCAAAGGAGAAUUGCCCUG
osa-miR399b  UGCCAAAGGAGAAUUGCCCUG
osa-miR399c  UGCCAAAGGAGAAUUGCCCUG
osa-miR399d  UGCCAAAGGAGAGUUGCCCUG
osa-miR399e  UGCCAAAGGAGAUUUGCCCAG
osa-miR399f  UGCCAAAGGAGAUUUGCCCAG
osa-miR399g  UGCCAAAGGAGAUUUGCCCAG
osa-miR399h  UGCCAAAGGAGACUUGCCCAG
osa-miR399i  UGCCAAAGGAGAGCUGCCCUG
osa-miR399j  UGCCAAAGGAGAGUUGCCCUA
osa-miR399k  UGCCAAAGGAAAUUUGCCCCG
             ********** *  *****
```

Supplementary Figure S3. Alignment of rice mature miR399 sequences. Conserved nucleotides are in black labeled with an asterisk (*). Mismatched nucleotides are indicated in colors (A, red; U, green; G, blue; C, purple). osa, *Oryza sativa.*
